# Metabolic effects of Tofogliflozin are efficiently enhanced with appropriate dietary carbohydrate ratio and are distinct from carbohydrate restriction

**DOI:** 10.14814/phy2.13642

**Published:** 2018-03-09

**Authors:** Shiori Ito, Toshio Hosaka, Wataru Yano, Takahiro Itou, Misako Yasumura, Yukari Shimizu, Hideyuki Kobayashi, Takashi Nakagawa, Keisuke Inoue, Sohei Tanabe, Takuma Kondo, Hitoshi Ishida

**Affiliations:** ^1^ Tokyo New Drug Research Laboratories Kowa Company, LTD. Tokyo Japan; ^2^ Third Department of Internal Medicine Division of Diabetes, Endocrinology and Metabolism Kyorin University School of Medicine Tokyo Japan

**Keywords:** Low‐carbohydrate diet, sodium‐glucose cotransporter 2 inhibitor

## Abstract

Sodium‐glucose cotransporter 2 inhibitors (SGLT2i) exert their antidiabetic effects by promoting urinary glucose excretion. Nutrition therapy is obviously important, but little is known about the interactions between SGLT2i agents and carbohydrate restriction. Therefore, we studied these interactions using an obese diabetic animal model. KK‐A^y^ mice were pair‐fed normal chow [NC; carbohydrate: fat: protein = 65:15:20], low carbohydrate [LC; 43:42:15] or severely carbohydrate restricted diets [SR; 12:45:43] for 12 weeks. Tofogliflozin (Tofo) was administered as the SGLT2i in the NC and LC diet groups. Blood glucose levels were significantly increased in the SR group. Tofo reduced blood glucose levels significantly in the NC group during the experiment and in the LC group at 2‐6 weeks. Plasma triglycerides were markedly elevated in the SR group without Tofo, but decreased in response to Tofo administration. Hepatic triglyceride contents were not changed by the LC or the SR diet alone. However, Tofo ameliorated hepatosteatosis in NC‐fed animals. Consistent with the downregulation of stearoyl‐CoA desaturase 1, the ratio of plasma monounsaturated to saturated fatty acids was significantly reduced in the LC with Tofo and in the SR alone groups, but was not altered in the NC with Tofo group. In summary, metabolism of glucose and lipids was improved by Tofo but not by the SR diet. Furthermore, Tofo improved these parameters more effectively in the NC than in the LC diet group. These data suggest that the effects of SGLT2i are distinct from those of carbohydrate restriction and that a nonrestricted dietary carbohydrate composition is essential for SGLT2i treatment to be effective.

## Introduction

Type 2 diabetes mellitus is characterized by a chronic hyperglycemic state due to decreased insulin sensitivity in target tissues, including skeletal muscle, adipocytes and the liver, and/or impairment of insulin secretion (Guillausseau and Laloi‐Michelin 2003; Stumvoll et al. 2007). Obesity is now a pandemic disease, with numerous adverse consequences, and is responsible for type 2 diabetes mellitus, hyperlipidemia, and hypertension (Bluher 2009). Elevated triglycerides (TG) exacerbate hyperglycemia through peripheral insulin resistance, ultimately resulting in cerebral infarction and cardiovascular disease (Mooradian 2009).

A class of antidiabetic drugs, the sodium‐glucose cotransporter 2 inhibitors (SGLT2i), reduces renal glucose reabsorption (Suzuki et al. 2012) and ameliorates hyperglycemia independently of insulin action (Whalen et al. 2015). These agents reportedly exert lipid lowering effects (Fujita and Inagaki 2014; Vallon and Thomson 2017), ameliorate steatosis (Hayashizaki‐Someya et al. 2015; Honda et al. 2016; Komiya et al. 2016), promote recovery of *β* cell function (Shimo et al. 2015), and prevent the development of atherosclerosis (Terasaki et al. 2015; Han et al. 2017).

Recently, several studies have demonstrated that a low‐carbohydrate (LC) diet has beneficial effects on obesity and glycemic control in the short‐term (Shai et al. 2008; Krebs et al. 2013). On the other hand, prospective cohort studies have shown severely carbohydrate restricted (SR) diets to be associated with increased rates of cardiovascular disease and mortality (Sjogren et al. 2010; Lagiou et al. 2012). In addition, long‐term LC diet intake reportedly leads to impaired glucose tolerance and insulin resistance in both normal and diabetic animal models (Handa et al. 2014). The LC diet was also reported to exert adverse vascular effects in atherosclerotic animal models (Foo et al. 2009; Handa et al. 2014).

However, whether or not the effectiveness of SGLT2i is influenced by the dietary carbohydrate ratio, especially by that of an LC diet, is still unclear. Moreover, whether or not the glucose losing effects of SGLT2i and carbohydrate restriction on metabolic homeostasis are similar also remains unknown. Therefore, in this study, metabolic homeostasis was investigated in KK‐A^y^ obese diabetic mice‐fed diets with different carbohydrate compositions, with and without SGLT2i administration.

## Materials and Methods

### Animals

Male KK‐A^y^ (KK‐A^y^/TaJcl) mice were purchased from CLEA Japan, Inc. (Tokyo, Japan) at 3 weeks of age. The mice were housed under a 12‐hour light/dark cycle (07:00/19:00) and given CE‐2 feed (CLEA Japan, Inc., Tokyo, Japan) and sterilized water ad libitum. Tofogliflozin (Tofo) was provided by Chugai Pharmaceutical Co., Ltd. (Tokyo, Japan). From the age of 6 weeks, KK‐A^y^ mice were pair‐fed calorie‐matched chow with normal compositions of carbohydrate [NC; carbohydrate (C): protein (P): fat (F) = 65:20:15], low carbohydrate (LC; C:P:F = 43:15:42) or severely restricted carbohydrate (SR; C:P:F = 12:43:45) (Research Diet Inc., New Brunswick, NJ) for 12 weeks. The NC and LC groups were given a diet containing 0.005% or 0.015% Tofo for 12 weeks. According to the plasma Tofo concentrations in mice and in humans, the 0.015% Tofo dose given to the KK‐A^y^ mice in this study is comparable to the therapeutic dose of 20 mg for patients with type 2 diabetes (Ishibashi et al. 2016). The animal care and experimental procedures were approved by the Animal Care Committee of Tokyo New Drug Research Laboratories, Kowa Company.

### Biochemical parameters

Blood samples were collected in the nonfasted state in the morning to measure plasma glucose and TG levels using assay kits from Wako Pure Chemical Industries (Osaka, Japan). Plasma insulin was measured employing a Mouse Insulin ELISA Kit (Shibayagi, Gunma, Japan). Plasma unsaturated and saturated fatty acid levels were measured using liquid chromatography–tandem mass spectrometry at CMIC Pharma Science Co., Ltd. (Tokyo, Japan).

### Oral glucose tolerance and insulin tolerance test

Oral glucose tolerance tests (OGTT) were performed as below. A glucose dose of 1 g/kg was administered orally to mice after an overnight fast. Blood samples were collected from the tail vein to measure blood glucose levels at the 0, 15, 30, 60, and 120 min time points. For insulin tolerance tests (ITT), mice were injected with 1 U/kg insulin intraperitoneally and blood glucose levels were measured at 0, 20, 40, 60, 80, 100, and 120 min.

### Liver tissue staining

Slices of liver tissue were immersed in phosphate buffered saline with 4% paraformaldehyde, and then embedded in paraffin. Deparaffinized sections (3 *μ*m thick) were stained with hematoxylin and eosin (HE). Frozen sections of liver tissue (7 *μ*m thick) were stained with Oil Red O.

### Hepatic TG levels

Four milliliters of chloroform: methanol (2:1) were added to 100 mg of liver tissue and stored at 4°C for 3 days and lipids were then extracted. Lipid extracts were dried and resuspended with 2‐propanol. TG levels in the lipid extracts were measured as described in the assay kit instructions and tissue lipid contents were then calculated.

### Gene expression analysis using real‐time quantitative PCR

For real‐time quantitative PCR (qPCR), an ABI 7900HT Fast Real‐Time PCR System (Thermo Fisher Scientific, Waltham, MA) was used with the following mouse probes: stearoyl‐CoA desaturase 1 (SCD1) (Mm00772290_m1), fatty acid synthase (FAS) (Mm00662319_m1), glucokinase (Gck) (Mm00439129_m1), phosphoenolpyruvate carboxykinase 1 (PEPCK) (Mm01247058_m1), glucose‐6‐phosphatase (Mm00839363_m1), carnitine palmitoyltransferase 1a (CPT1a) (Mm01231183_m1), FGF21 (Mm00840165_g1), acyl‐CoA oxidase 1 (ACO) (Mm01246831_m1), TNF*α* (Mm00443258_m1), hormone sensitive lipase (HSL) (Mm00495359_m1), and *β*‐actin (Mm02619580_g1).

### Statistical analysis

The results are presented as means ± standard deviation (SD). Differences between groups were examined for statistical significance using *Dunnett's test*. In all analyses, *P < 0.05* was taken to indicate statistical significance.

## Results

### Tofo treatment improved metabolic parameters while carbohydrate restriction did not

**Figure 1:**
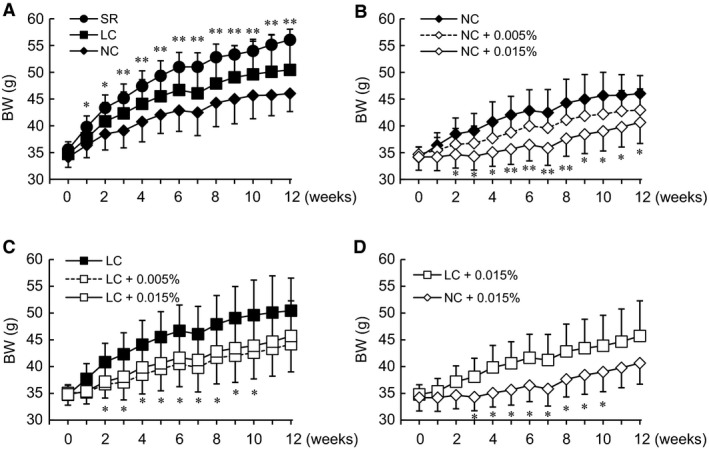
Body weights of KK‐A^y^ mice with carbohydrate restriction and Tofo treatment for 12 weeks. Body weights (BW) of KK‐A^y^ mice with carbohydrate restriction (A), with Tofo treatment while fed the NC (B) or the LC (C) diet and with Tofo treatment on both diets (D) are shown as means ± SD (*n* = 6–8). *, **, *P* < 0.05, 0.01 versus NC or untreated group by Dunnett's test.

To compare the effects of SGLT2i and carbohydrate restriction on metabolic homeostasis, body weights were determined in KK‐A^y^ mice in the NC and LC groups, which received Tofo, and mice in the SR group, which did not. Body weights were significantly increased in the SR group after one week and tended to rise in the LC as compared to the NC group (Fig. 1A), despite caloric intakes being matched among the groups. On the other hand, the Tofo‐treated groups showed decreased body weights as compared to the untreated groups (Fig. 1B, C), and, surprisingly, the impact of Tofo on body weight was more marked in the NC‐ than in the LC‐fed mice, and reached statistical significance during the period from 3 to 10 weeks. (Fig. 1D). Body compositions were investigated after the feeding experiment. Lean mass was estimated based on a calculation employing body weight and several adipose tissue weights in KK‐A^y^ mice (Table 1). The SR group exhibited a significant increase in body weight and lean mass, suggesting an effect of the high‐protein content of the SR diet. Subcutaneous adipose tissue weight also tended to be increased as compared to that of the NC group. The increased body weight in the SR group might be involved in determining both fat and lean masses.

**Table 1:** Body weight, visceral and subcutaneous adipose tissue, and estimated lean mass in KK‐A^y^ mice receiving diets with different nutrient compositions and Tofo treatment

	Body weight (g)	Visceral fat (g)	Subcutaneous fat (g)	Lean mass (g)
NC	45.8 ± 3.7	3.5 ± 0.5	2.8 ± 0.5	39.5 ± 3.1
NC + 0.005%	42.9 ± 1.5	3.7 ± 0.3	2.2 ± 0.5	37.0 ± 1.7
NC + 0.015%	41.1 ± 3.5	3.5 ± 0.3	1.9 ± 0.4#	35.7 ± 3.1
LC	49.9 ± 5.1	3.4 ± 0.4	3.5 ± 1.1	43.0 ± 4.0
LC + 0.005%	44.8 ± 6.3	4.1 ± 0.4	2.8 ± 1.1	37.8 ± 5.1
LC + 0.015%	47.7 ± 8.7	4.4 ± 0.9	3.4 ± 1.3	39.9 ± 6.8
SR	54.6 ± 1.9**	3.6 ± 0.7	4.1 ± 1.0	46.8 ± 2.0**

Visceral fat contains epididymal, mesenteric and retroperitoneal adipose tissues. Lean mass was estimated based on a calculation employing body weight, and both visceral and subcutaneous fat weights. Mean ± SD (*n* = 4–8). **P < 0.01 versus NC; ^#^ *P* < 0.05 versus untreated group by Dunnett's test.

**Figure 2:**
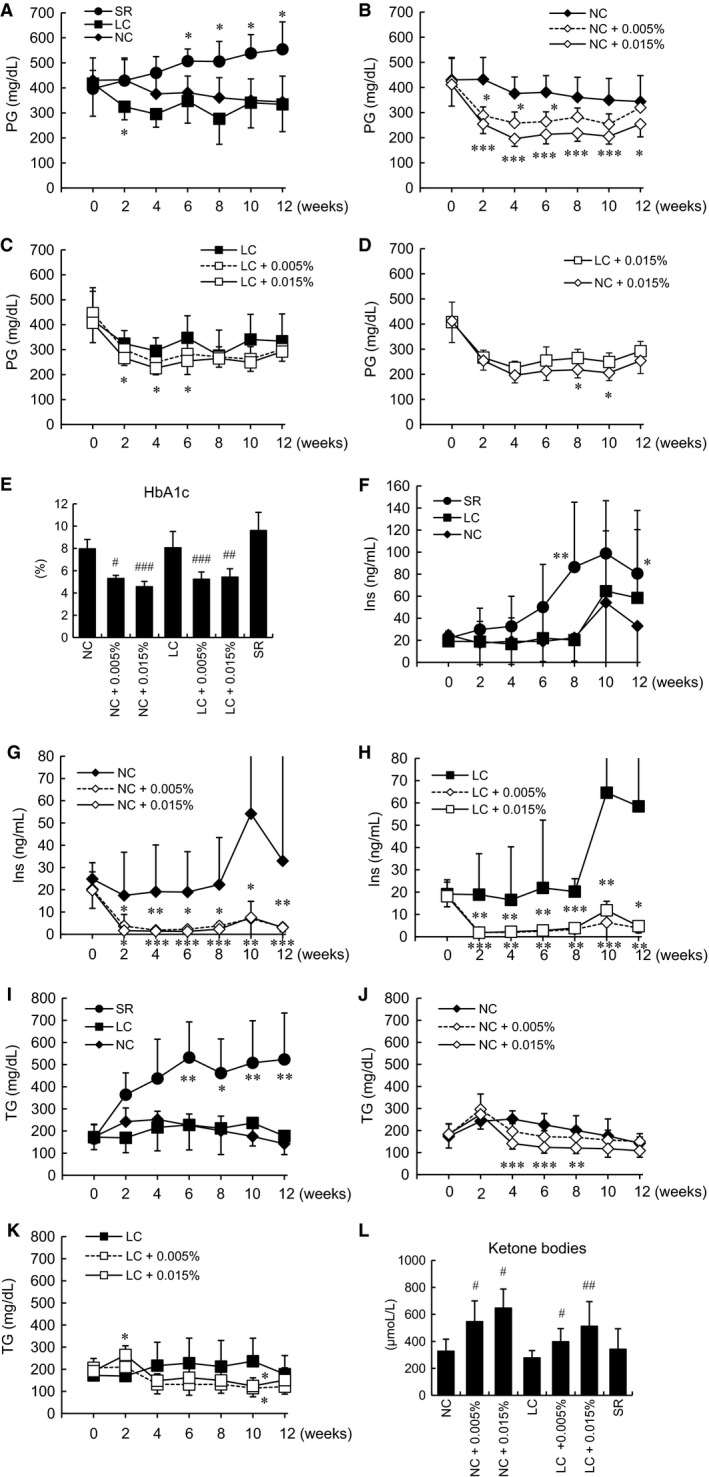
Plasma blood glucose, insulin and triglyceride levels of KK‐A^y^ mice with carbohydrate restriction and Tofo treatment. The serum plasma glucose (PG), insulin (Ins) and triglyceride (TG) levels in KK‐A^y^ mice with carbohydrate restriction (A, F, I), in Tofo‐treated mice fed the NC diet (B, G, J), the LC diet (C, H, K), and administered Tofo on both diets (D), are shown as means ± SD (*n* = 6–8). *, **, ***, *P* < 0.05, 0.01, 0.001 versus NC or untreated group by Dunnett's test. HbA1c (E) and plasma ketone body levels (L) are shown as means + SD (*n* = 6–8). #, ##, ###, *P* < 0.05, 0.01, 0.001, versus untreated group by Dunnett's test.

**Figure 3:**
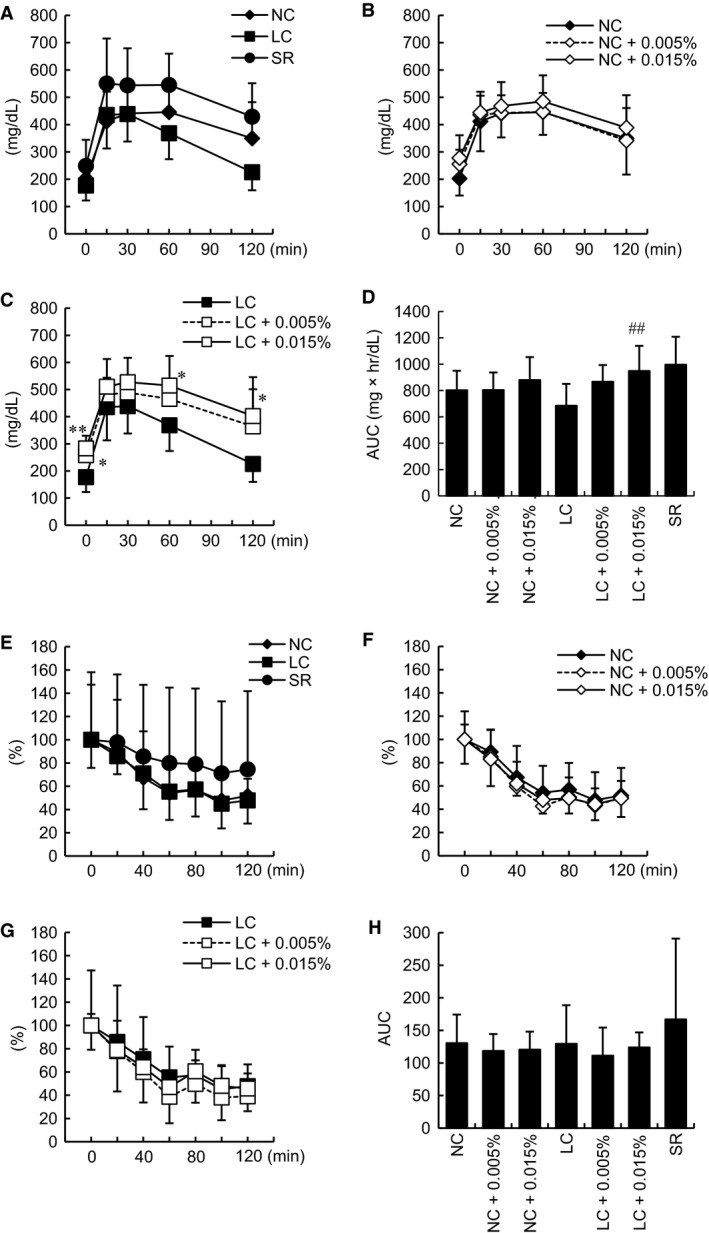
Oral glucose and insulin tolerance tests in KK‐A^y^ mice with carbohydrate restriction and Tofo treatment. Blood glucose levels on oral glucose tolerance tests of KK‐A^y^ mice with carbohydrate restriction (A), mice with Tofo treatment fed the NC diet (B) and the LC diet (C), and area under curve (AUC) values for all groups (D), are shown as means ± SD (*n* = 6–8). Blood glucose changes from baseline and AUC for the insulin tolerance test are also shown (E–H) as means ± SD (*n* = 6–8).

Next, plasma metabolic parameters were investigated. In the SR group, blood glucose tended to be elevated after 4 weeks and was significantly increased from 6 weeks until the end of the observation period as compared to the NC group (Fig. 2A). The LC diet did not markedly decrease blood glucose levels as compared to the NC diet (Fig. 2A), while Tofo treatment reduced blood glucose as compared to the levels in untreated animals, with the differences being significant up to 12 weeks in the NC‐fed and from 2 to 6 weeks in LC‐fed mice (Fig. 2B, C). Moreover, the effect of Tofo on blood glucose levels in animals on the LC diet was slightly attenuated as compared to those observed in NC‐fed mice (Fig. 2D). After the 12‐week Tofo treatment, HbA1c levels were significantly reduced in the NC and LC groups (Fig. 2E). Plasma insulin levels were markedly increased in the SR group (Fig. 2F), while being significantly decreased with Tofo administration in both the NC and the LC group (Fig. 2G, H). Plasma TG levels rose significantly in the SR group during the experiments (Fig. 2I), but were reduced in response to Tofo under both NC and LC feeding conditions (Fig. 2J, K). No significant difference was observed in plasma insulin or TG levels between the NC and LC groups after Tofo treatment. Plasma concentrations of ketone bodies were significantly increased in the NC and LC groups receiving Tofo treatment (Fig. 2L). On GTT (Fig. 3A–D), the SR group tended to show higher blood glucose levels, but no significant alteration of blood glucose was observed with Tofo treatment in mice on the NC diet. In the LC with Tofo group, blood glucose levels were already significantly elevated at the start of GTT. Ketone bodies were increased in the NC and LC mice receiving Tofo treatment in this study and, furthermore, a previous study also demonstrated an increase in ketone bodies and the upregulation of hepatic glucose production in response to Tofo treatment (Obata et al. 2016). Although gluconeogenesis was also previously demonstrated to be induced as compensation for the urinary glucose excretion promoted by SGLT2i (Chiba et al. 2016; Komiya et al. 2016; Obata et al. 2016), further investigation is necessary to address whether gluconeogenesis might be associated with Tofo treatment in this study. On ITT (Fig. 3E–H), no amelioration of insulin resistance was observed in any of the groups. Considering that KK‐A^y^ mice are an established model of diabetes with severe obesity, insulin resistance, and hyperglycemia, the effects of SGLT2i might be masked due to the high blood glucose levels of KK‐A^y^ mice.

Our observations suggest that metabolic parameters might be improved by Tofo treatment, though not when administered in conjunction with a carbohydrate restricted diet, in KK‐A^y^ mice. Furthermore, the effects of Tofo appear to be more efficiently enhanced by NC than by LC feeding conditions.

### The hepatic steatosis‐ameliorating effects of Tofo are potentiated under appropriate carbohydrate feeding conditions

**Figure 4:**
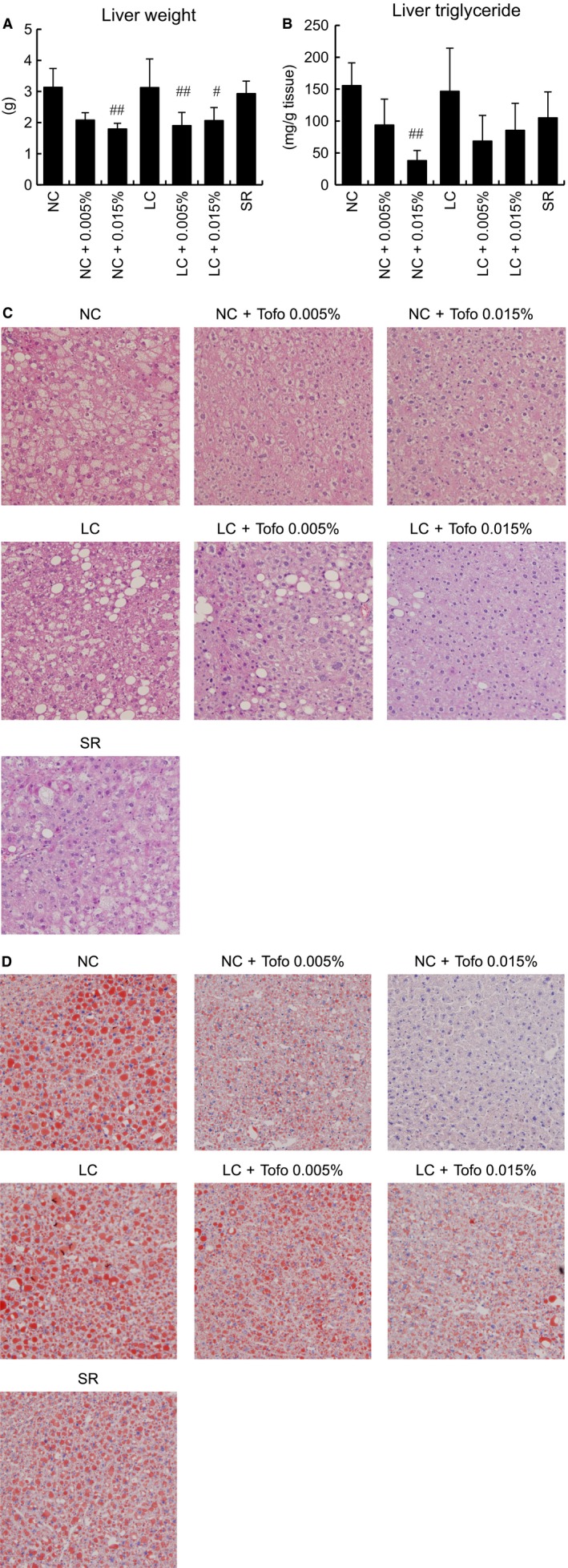
Characterization of liver findings in KK‐A^y^ mice receiving carbohydrate‐restricted diets and Tofo treatment. Liver weight (A) and TG content (mg per gram) of hepatic tissue (B) after 12‐week treatment are shown as means + SD (*n* = 4–8). #, ## *P* < 0.05, 0.01 versus untreated group by Dunnett's test. Hepatic tissue was stained with HE (C) and oil red O (D). The magnification is 100x.

**Figure 5:**
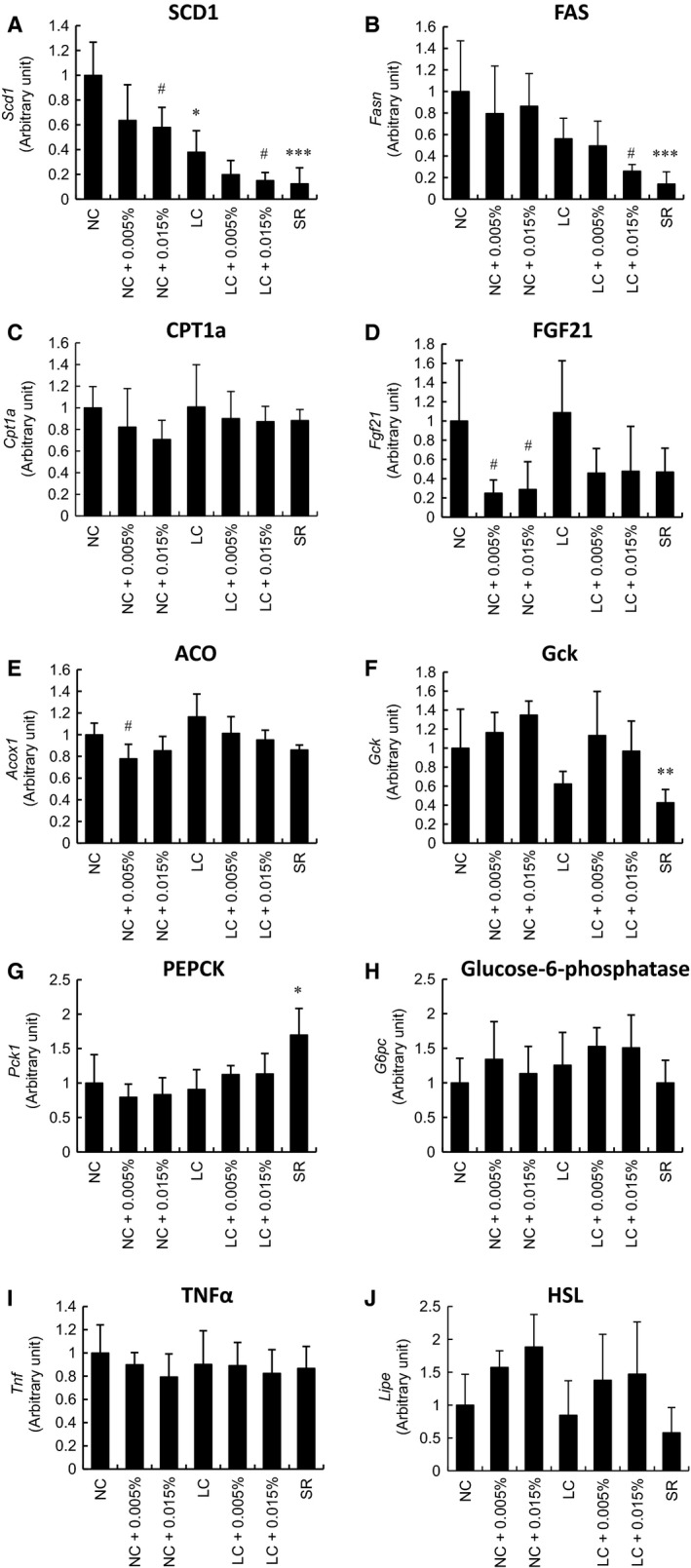
Hepatic and adipose mRNA expressions in KK‐A^y^ mice receiving carbohydrate restriction and Tofo treatment. Hepatic mRNA expression levels of SCD1 (A), FAS (B), CPT1a (C), FGF21 (D), ACO (E), Gck (F), PEPCK (G) and glucose‐6‐phosphatase (H) are shown as means + SD, (*n* = 4–8). TNF *α* (I) and HSL (J) expression levels in epididymal adipose tissue are shown as means + SD, (*n* = 4–8). *, **, ***, *P* < 0.05, 0.01, 0.001 versus NC; #, *P* < 0.05, versus untreated group by Dunnett's test.

To clarify the mechanisms by which different metabolic changes develop under each feeding condition, hepatic phenotypes were examined. Liver weight was lower in animals receiving diets with Tofo, while carbohydrate restriction had no effect on liver weight (Fig. 4A). TG deposition in the liver was significantly decreased in the NC with Tofo group, while no significant change was detected in any of the other groups (Fig. 4B). HE and oil red O staining revealed that steatosis in the NC and LC groups was ameliorated by Tofo, and that Tofo treatment markedly reduced lipid accumulation in NC‐ as compared to LC‐fed mice (Fig. 4C, D), with no clear changes in the LC or SR group as compared to the NC group (Fig. 4C, D). As regards gene expressions related to fatty acid metabolism in the liver, SCD1 was significantly reduced after Tofo treatment. The downregulation of SCD1 also depended on dietary carbohydrate contents (Fig. 5A). The expression of FAS, which is involved in fatty acid synthesis, was decreased in the LC with Tofo group and in the SR group not given Tofo (Fig. 5B). The gene expressions of CPT1a, the rate‐limiting enzyme of *β* oxidation in the liver, did not differ among the groups (Fig. 5C). The FGF21 expression level was significantly decreased in the Tofo‐treated NC group, and tended to be reduced in the LC group receiving Tofo and SR alone group (Fig. 5D). In addition, we investigated the hepatic expression level of ACO which is known to be an important factor in fatty acid oxidation (Fig. 5E). Even though the only significant change observed was in the NC with low‐dose Tofo group, this alteration of the ACO expression level was apparently small as compared to that of the FGF21 expression level. Next, expressions of genes which regulate glycolysis and gluconeogenesis were investigated. The expression of Gck was downregulated only in the SR alone group. Glucose‐6‐phosphatase was unchanged but PEPCK was significantly upregulated (Fig. 5F–H), indicating that hepatic glucose production was enhanced by the SR diet. Gene expressions in epididymal adipose tissue were also investigated. None of the groups showed a change in the TNF*α* expression level, while HSL expression tended to be increased in the Tofo‐treated NC and LC groups (Fig. 5I, J). Thus, Tofo might most effectively ameliorate hepatosteatosis, in KK‐A^y^ mice, when given with the NC diet.

### Appropriate dietary carbohydrate ratio is important for raising the ratio of plasma unsaturated to saturated fatty acids

**Figure 6:**
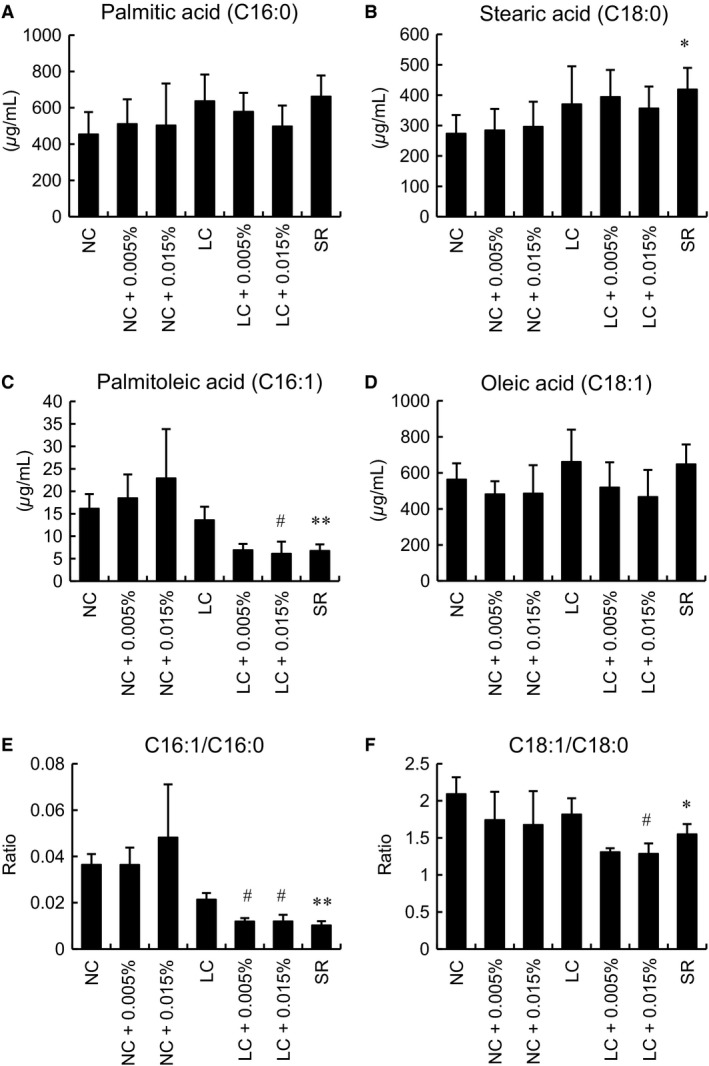
Plasma fatty acid levels of KK‐A^y^ mice with carbohydrate restriction and Tofo treatment. Plasma palmitic acid (C16:0) (A), stearic acid (C18:0) (B), palmitoleic acid (C16:1) (C) and oleic acid (C18:1) (D), after 12 weeks of Tofo treatment, and the C16:1/C16:0 (E) and C18:1/C18:0 (F) ratios are shown as means + SD, (*n* = 4–5). *, **, *P* < 0.05, 0.01 versus NC; #, *P* < 0.05 versus untreated group by Dunnett's test.

We focused on plasma fatty acids to elucidate the factors affecting glucose and lipid homeostasis under each diet and treatment condition. Palmitic acid (C16:0) tended to be increased in the LC and SR as compared to the NC group (Fig. 6A). Stearic acid (C18:0) was also apparently increased in the LC group, while being significantly increased in the SR group, as compared to the NC group (Fig. 6B). Palmitoleic acid (C16:1) was significantly decreased in the SR group as compared to the NC group (Fig. 6C). Tofo treatment with the LC diet influenced the level of palmitoleic acid (Fig. 6C). The C16:1/C16:0 ratio was significantly lower in the SR group than in the NC group, as was observed in the LC with Tofo group but not in the NC with Tofo group (Fig. 6E, F). The unsaturated to unsaturated fatty acid ratio might thus be decreased by Tofo with the LC diet, as well as with the SR diet, through the downregulation of SCD1.

## Discussion

In this study, we examined KK‐A^y^ obese diabetic mice focusing on metabolic homeostasis, using diets with different carbohydrate compositions administered with the SGLT2i Tofo. We investigated the interactions of SGLT2i and carbohydrate restriction, and compared the metabolic effects among these treatment combinations, especially those involving severe carbohydrate restriction. Considering nutritional therapy in a real world setting, carbohydrate restriction is assumed to be associated with substantial increases in dietary fat and protein compositions. Therefore, in this study, we created several diets with different compositions but the same number of calories and compared the metabolic effects on KK‐A^y^ mice with similar calorie intakes achieved by pair‐feeding.

First, we investigated the efficacies of Tofo plus diets with different carbohydrate compositions. Tofo administration was associated with reduced body weight gain and blood glucose levels in both the NC and the LC diet group, consistent with previous reports showing that SGLT2i treatment prevented the development of obesity and diabetes in mice‐fed standard chow with a high‐fat content (Atageldiyeva et al. 2016; Komiya et al. 2016; Obata et al. 2016). However, our data further demonstrated that Tofo treatment more effectively reduced weight and blood glucose when given with NC than with LC, and the difference was statistically significant. In terms of lipid metabolism, several reports have described SGLT2i as preventing the liver steatosis in NASH (nonalcoholic steatohepatitis) model mice (Hayashizaki‐Someya et al. 2015; Honda et al. 2016) and obese diabetic animals (Komiya et al. 2016; Nishimura et al. 2016). In the present study, Tofo decreased hepatic TG contents under both conditions, and the effect was enhanced in NC‐fed as compared to LC‐fed mice. These data suggest the efficacy of SGLT2i to be affected by the carbohydrate composition of the diet.

Next, we investigated the effects of carbohydrate restriction on glucose and lipid metabolism. In the SR group, body weight, blood glucose, and insulin levels were significantly increased as compared to the NC group. Plasma TG was markedly elevated in response to the SR diet, leading to insulin resistance. Despite maintenance of the same caloric intake by pair feeding, the SR diet worsened plasma glucose and lipid levels as compared to the NC diet in an obese diabetic animal model. Since the SR diet consists of severely restricted carbohydrate and a high‐protein content, it is also possible that the large amount of protein consumed might have affected the metabolic processes of our KK‐A^y^ mice. A number of earlier reports described suppression of body weight gain with a high‐protein diet (Garcia‐Caraballo et al. 1832; Petzke et al. 2014), while other prior studies demonstrated a low‐protein diet to reduce body weight gain mediated by energy expenditure *via* the upregulation of FGF21 (Laeger et al. 2014). The data of the previous studies were obtained using nonobese mice. Although the effect of a high‐protein diet on metabolism is still controversial, our data showed a reduction in the FGF21 expression level in pair‐fed KK‐A^y^ mice receiving Tofo treatment and in those on the SR diet without Tofo administration. Unexpectedly, we found CPT1 and ACO expressions to apparently be unchanged. Thus, it is possible that the Tofo treatment given in this study might have had only a minimal impact on the fatty acid oxidation in the liver. On the other hand, while we did not conduct examinations using indirect calorimetry in this study, Tofo treatment significantly increased plasma ketone bodies and tended to upregulate HSL in adipose tissue. Previous reports described Tofo treatment as decreasing the respiratory quotient, indicating a metabolic shift (Suzuki et al. 2014; Obata et al. 2016), and activating the protein kinase A pathway involved in HSL in adipose tissue which was partly mediated by a liver‐brain‐adipose neural axis (Sawada et al. 2017). These findings suggest upregulation of fatty acid oxidation at the whole body level in response to Tofo treatment. Epididymal fat tissue weight tended to be decreased with carbohydrate restriction and slightly increased in Tofo‐treated groups. The TNF*α* expression level was slightly downregulated in response to Tofo treatment. This observation is consistent with those of a previous study demonstrating that SGLT2i prevent ectopic fat accumulation in the liver (Komiya et al. 2016). The phenomena observed in the present study may reflect dietary nutrient compositions. Furthermore investigations, focusing on several distinct tissue types, might facilitate elucidation of the mechanisms by which SGLT2i exert their beneficial actions.

Although SGLT2i treatment and carbohydrate restriction both aim to favorably regulate glucose metabolism, our results revealed the opposite outcome. Body weight gain was decreased with Tofo, but was increased in animals on the SR diet. Plasma glucose and insulin levels were reduced by Tofo treatment, but neither the LC nor the SR diet. KK‐A^y^ mice also exhibited a significant HbA1c reduction in response to Tofo treatment, but not to carbohydrate restriction, suggesting a therapeutic effect of Tofo on glycemic control. Atageldiyeva et al. (Atageldiyeva et al. 2016) subjected nonobese mice to carbohydrate restriction and SGLT2i treatment and reported that body weight gain increased on a low‐carbohydrate diet and decreased with SGLT2i treatment, which is consistent with the results obtained in our present study with KK‐A^y^ mice. The nonobese mice in the study of Atageldiyeva and colleagues showed no blood glucose level change, but the SGLT2i suppressed blood glucose elevation only with oral glucose loading. Considering the mechanism by which SGLT2i excrete excess blood glucose *via* the urine, it is reasonable to speculate that the SGLT2i effect would be highly apparent in a hyperglycemic model such as KK‐A^y^ mice. The expression of Gck, which is involved in glycolysis, tended to increase in the Tofo‐treated groups, but decreased significantly in response to the SR diet. Gluconeogenic genes such as PEPCK and glucose‐6‐phosphatase showed no significant changes with Tofo administration, but hepatic PEPCK induction was detected in the SR group. Similar results were obtained for lipid metabolism. Tofo reduced plasma TG levels and reduced lipid accumulation in the liver, while the SR diet increased plasma TG markedly without improving hepatic TG. Tofo‐treated mice showed a significant increase in ketone bodies in response to the NC and LC diets, but not to the level of ketoacidosis. Treatment with Tofo may induce urinary glucose excretion to suppress peripheral glucose utilization, and plasma free fatty acids might be metabolized in the liver as an energy source, thereby resulting in ketone body production. Therefore, it is possible that a metabolic shift from carbohydrate to fatty acid oxidation occurred in the mice in this study, as well as in a previous study using DIO and KK‐A^y^ mice (Suzuki et al. 2014). These data clearly show SGLT2i treatment and carbohydrate restriction to exert quite different effects, presumably by different mechanisms, on metabolic homeostasis.

On the other hand, some of the responses obtained with the interventions in this study were in the same direction, that is, they did not differ between SGLT2i treatment and carbohydrate restriction. FAS and SCD1, both of which are involved in lipid metabolism, were significantly downregulated in both the LC with Tofo and the SR diet alone groups. SCD1 is an enzyme which converts saturated fatty acids into monounsaturated fatty acids (Ntambi 1992). In this study, due to the SCD1 reduction, the ratio of unsaturated to saturated fatty acids was significantly decreased, to the same level, in both groups. Reportedly, monounsaturated fatty acids exert beneficial effects against metabolic syndrome (Gillingham et al. 2011). Despite the parallel effects on FAS, SCD1 and unsaturated fatty acids, our results revealed different outcomes in terms of glucose and lipid metabolism. Specifically, we observed beneficial effects in the LC with Tofo group and unfavorable effects in the SR diet alone group. Carbohydrate restricted diets are relatively high in fat. Under LC‐ or SR‐fed conditions, an adaptive response against increasing fat intake might occur due to the suppression of fatty acid synthesis *via* the SREBP‐1 transcription factor which regulates FAS and SCD1. The downregulation of SCD1 might then lower the unsaturated fatty acid level, leading to unfavorable effects on metabolism. Decreased SCD1 expression is reportedly related to fatty liver development, subsequently leading to insulin resistance (Ntambi 1992; Stefan et al. 2008; Fernandez Gianotti et al. 2013; Geng et al. 2013). In contrast, although SCD1 expression was reduced in this study, obesity and lipid metabolism showed improvements in Tofo‐treated mice. In this study, the actions of Tofo apparently overcame the unfavorable metabolic effects of the LC diet. Liver CPT1a was not altered in the Tofo‐treated groups, though HSL tended to be increased in the epididymal fat of mice receiving Tofo, possibly reflecting fatty acid oxidation. In mice on the NC diet, Tofo did not change the unsaturated to saturated fatty acid ratios and the metabolic effects of Tofo showed no attenuation, in contrast to the LC diet with Tofo. Although SCD1 expression was decreased in the NC with Tofo group, the unsaturated to saturated fatty acid ratio was not altered. However, the SCD1 expression level was not markedly reduced in the NC as compared to the LC with Tofo group or the SR alone group, in which the unsaturated to saturated fatty acid ratios were significantly decreased. Therefore, SCD1 expression levels were suggested to possibly be pivotal in determining unsaturated to saturated fatty acid ratio changes. These data raised the possibility that the carbohydrate composition of the NC diet, but not that of the LC diet, might be very important for an optimally effective response to Tofo.

These observations taken together indicate that, unlike a carbohydrate restricted diet, SGLT2i might have beneficial effects on glycemic control and lipid metabolism, particularly when administered with appropriate dietary carbohydrate compositions.

## Conflict of Interest

SI, WY, TI, MY, YS, HK, TN, KI, and ST are employees of Kowa Company, Ltd. None of the authors have any financial interests related to this work.
